# Phenethylamine is a substrate of monoamine oxidase B in the paraventricular thalamic nucleus

**DOI:** 10.1038/s41598-021-03885-6

**Published:** 2022-01-07

**Authors:** Youhei Obata, Mie Kubota-Sakashita, Takaoki Kasahara, Masafumi Mizuno, Takahiro Nemoto, Tadafumi Kato

**Affiliations:** 1 Department of Neuropsychiatry, Toho University Graduate School of Medicine, Tokyo, Japan; 2 Laboratory for Molecular Dynamics of Mental Disorders, Center for Brain Science, RIKEN, Wako-shi, Saitama, Japan; 3 Department of Psychiatry, Faculty of Medicine, Juntendo University, Tokyo, Japan; 4 Career Development Program, Center for Brain Science, RIKEN, Wako-shi, Saitama, Japan; 5 Tokyo Metropolitan Matsuzawa Hospital, Tokyo, Japan

**Keywords:** Neuroscience, Diagnostic markers, Bipolar disorder

## Abstract

Monoamine oxidase (MAO) is a key enzyme responsible for the degradation of neurotransmitters and trace amines. MAO has two subtypes (MAO-A and MAO-B) that are encoded by different genes. In the brain, MAO-B is highly expressed in the paraventricular thalamic nucleus (PVT); however, its substrate in PVT remains unclear. To identify the MAO-B substrate in PVT, we generated *Maob* knockout (KO) mice and measured five candidate substrates (i.e., noradrenaline, dopamine, 3-methoxytyramine, serotonin, and phenethylamine [PEA]) by liquid chromatography tandem mass spectrometry. We showed that only PEA levels were markedly elevated in the PVT of *Maob* KO mice. To exclude the influence of peripheral MAO-B deficiency, we developed brain-specific *Maob* KO mice, finding that PEA in the PVT was increased in brain-specific *Maob* KO mice, whereas the extent of PEA increase was less than that in global *Maob* KO mice. Given that plasma PEA levels were elevated in global KO mice, but not in brain–specific KO mice, and that PEA passes across the blood–brain barrier, the substantial accumulation of PEA in the PVT of *Maob* KO mice was likely due to the increase in plasma PEA. These data suggest that PEA is a substrate of MAO-B in the PVT as well as other tissues.

## Introduction

**Figure 1:**
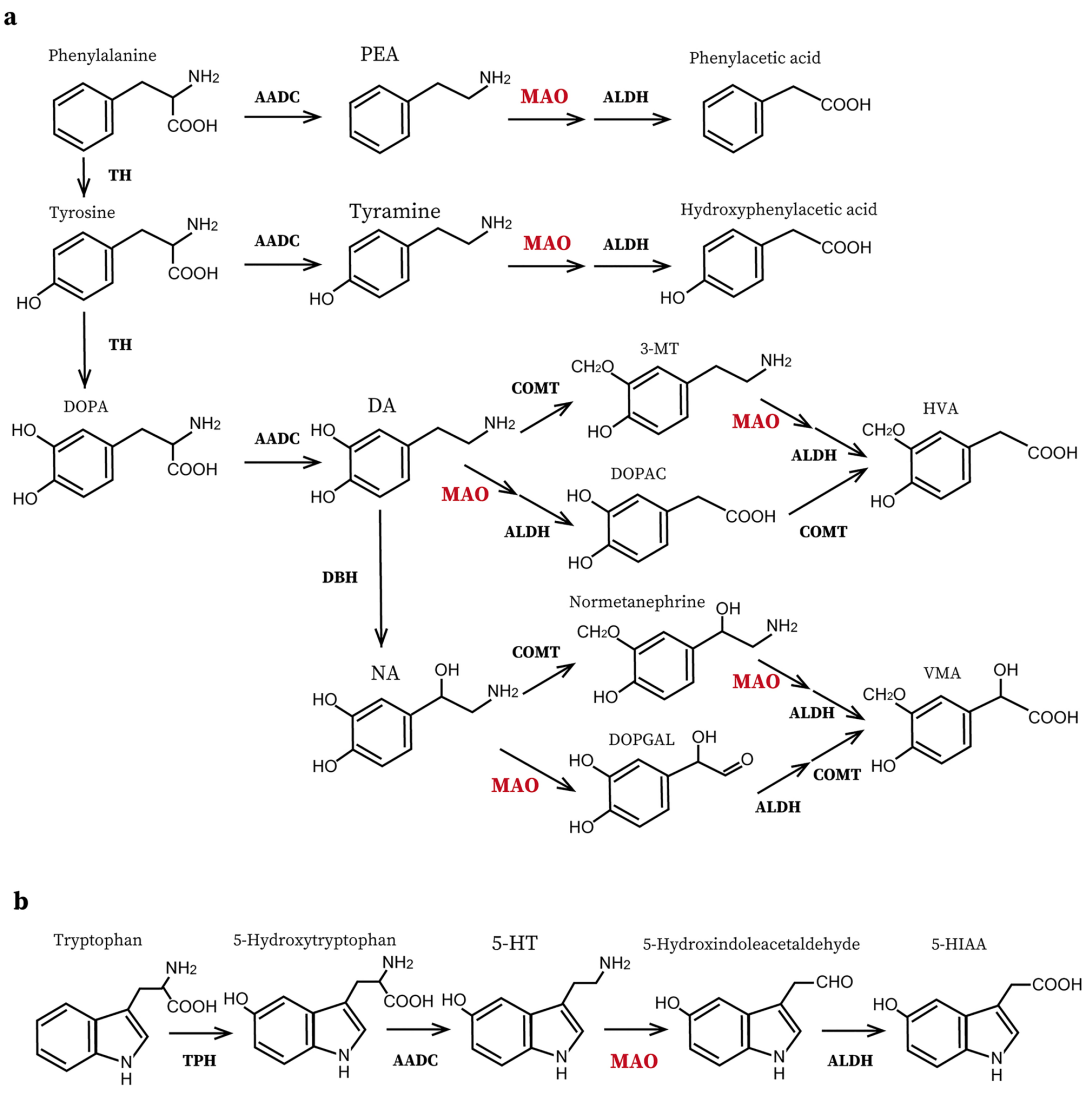
The MA metabolic pathways in the brain. (**a**) The synthetic and degradation pathways of phenethylamine (PEA), tyramine, dopamine (DA), and noradrenaline (NA). (**b**) The metabolic pathway of serotonin (5-HT). *AADC* aromatic l-amino acid decarboxylase, *ALDH* alcohol dehydroxylase, *COMT* catechol *O*-methyltransferase, *DBH* dopamine β-dehydroxylase, *DOPAC* 3,4-dihydroxyphenylacetic acid, *DOPGAL* 3,4-dihydroxyphenylglycolaldehyde, *5-HIAA* 5-hydroxyindole acetic acid, *HVA* homovanillic acid, *3-MT* 3-methoxytyramine, *TH* tyrosine hydroxylase, *TPH* tryptophan hydroxylase, *VMA* vanillylmandelic acid.

The monoamine (MA) neurotransmission system is important for a broad range of brain functions and dysfunctions, including neuropsychiatric disorders. MAs are degraded by monoamine oxidases (MAOs). Two types of MAOs (MAO-A and MAO-B) have been identified, the genes of which are located on the X chromosome and with a tail-to-tail arrangement^1^. MAOs were originally characterized using selective antagonists: MAO-A, inhibited by clorgyline, mainly metabolizes 5-hydroxytryptamine (5-HT), noradrenaline (NA), and dopamine (DA); and MAO-B, which is sensitive to selegiline, displays a higher affinity for phenethylamine (PEA) and DA (Fig. 1)^1–3^. MAOs are expressed predominantly in specific regions of the central nervous system and peripheral tissues. In the brain, MAO-A is highly expressed in noradrenergic neurons (e.g., the locus coeruleus)^4–6^, and MAO-B is dominant in serotonergic neurons (e.g., the dorsal raphe nucleus [DR]) and the paraventricular thalamic nucleus (PVT)^4–7^, whereas it also plays a role in glial cells^8–10^.

The PVT is one of the midline and intralaminar groups of thalamic nuclei and originates as part of the epithalamus. The relatively small nucleus extends along the anteroposterior axis and connects to regions that are important for emotion regulation, such as the medial prefrontal cortex, the bed nucleus of the stria terminalis, the nucleus accumbens (NAc), the insular cortex (Ins), and the amygdala^11–13^. Recently, PVT was implicated in stress response^14–17^, addiction-related behavior^11,18^, and fear conditioning^19^. Moreover, we reported that neural dysfunction of the PVT could lead to spontaneous and recurrent depression-like episodes in mice^20^, which was indicative of a pivotal role of the PVT in the stress response and emotional behaviors of animals.

According to a recent search of a comprehensive database (https://mouse.brain-map.org/experiment/show/71670489), MAO-B is highly expressed in the PVT. Because no signal was detected in the PVT by in situ hybridization for MA synthases, it is speculated that MAs are not synthesized in the PVT. The PVT is innervated by serotonergic neurons from the DR and median raphe nucleus^11,12,21^ and noradrenergic neurons from the locus coeruleus^17,21^. Indeed, 5-HT_7_ receptors and α_2B_ adrenoceptors are highly expressed in the PVT^22,23^, and 5-HT and NA are particularly abundant in the PVT^24^. Generally, however, the preferential substrate of MAO-B is neither 5-HT nor NA but rather DA or PEA. DA D2 receptors are expressed in the posterior PVT^11,17^, and if DA modulates neurotransmission in the PVT region, there should be projections from DA neurons; however, there are no reported projections from the ventral tegmental area (VTA), which is the main origin of DA release^21,25^. Although dopaminergic neurons are also thought to produce PEA, PEA neurotransmission in the PVT remains unknown. Thus, the physiological role of MAO-B in the PVT, including the MAO-B substrate in this region, is enigmatic. It is important to identify the substrate of MAO-B in the PVT in order to understand the biological rationale for the high expression of MAO-B in this region.

MAO-B substrates have been elucidated by determining the oxidation reaction of MAs in the presence of MAO-A inhibitors or measuring the elevation of endogenous MA concentrations following administration of MAO-B inhibitors^1–3,26^. However, “selective” inhibition of MAO-A or MAO-B is not precisely specific to each isoenzyme, and the inhibitors potentially act on mechanisms other than MAO inhibition. A gene knockout (KO) technique using the CRISPR/Cas9 system can be a more rigorous approach to identifying MAO-B substrates. Previous studies reported elevated PEA levels in the brain of *Maob* KO mice^27,28^, although they used whole-brain samples or bulk tissues. PEA is synthesized in DA neurons^29^ and so-called “D-cells” or “D-neurons” that are immunoreactive for aromatic l-amino acid decarboxylase but contain neither tyrosine hydroxylase nor serotonin^30,31^. PEA, as well as tyramine, tryptamine, and octopamine, is called a “trace amine”, and its concentration in the brain is several 100-fold lower than those of classical MA neurotransmitters^29,32–34^. It is technically challenging to measure PEA levels in a small nucleus and, to the best of our knowledge, there are no reports on measurement of PEA concentration in the PVT. Furthermore, PEA is reportedly present in various food items and produced by bacteria, including gut microbiota^35–38^. These peripheral PEAs are absorbed into the bloodstream and readily pass across the blood–brain barrier (BBB)^39–42^.

In this study, we focused on the PVT while also targeting the seven other brain regions and successfully measured levels of PEA and other MAs in small areas, including the PVT, using advanced high-performance liquid chromatography coupled with tandem mass spectrometry (LC–MS/MS) capable of highly sensitive measurements with small sample amounts. The concentrations of PEA and other MAs in the PVT were compared between wild-type (WT) mice and *Maob* KO mice in order to identify the MAO-B substrate in the PVT. Additionally, we developed brain-specific *Maob* KO mice, as well as global *Maob* KO mice, based on the ability of peripheral PEA to pass across the BBB.

## Results

### Generation of Maob KO mice and Maob flox mice

**Figure 2:**
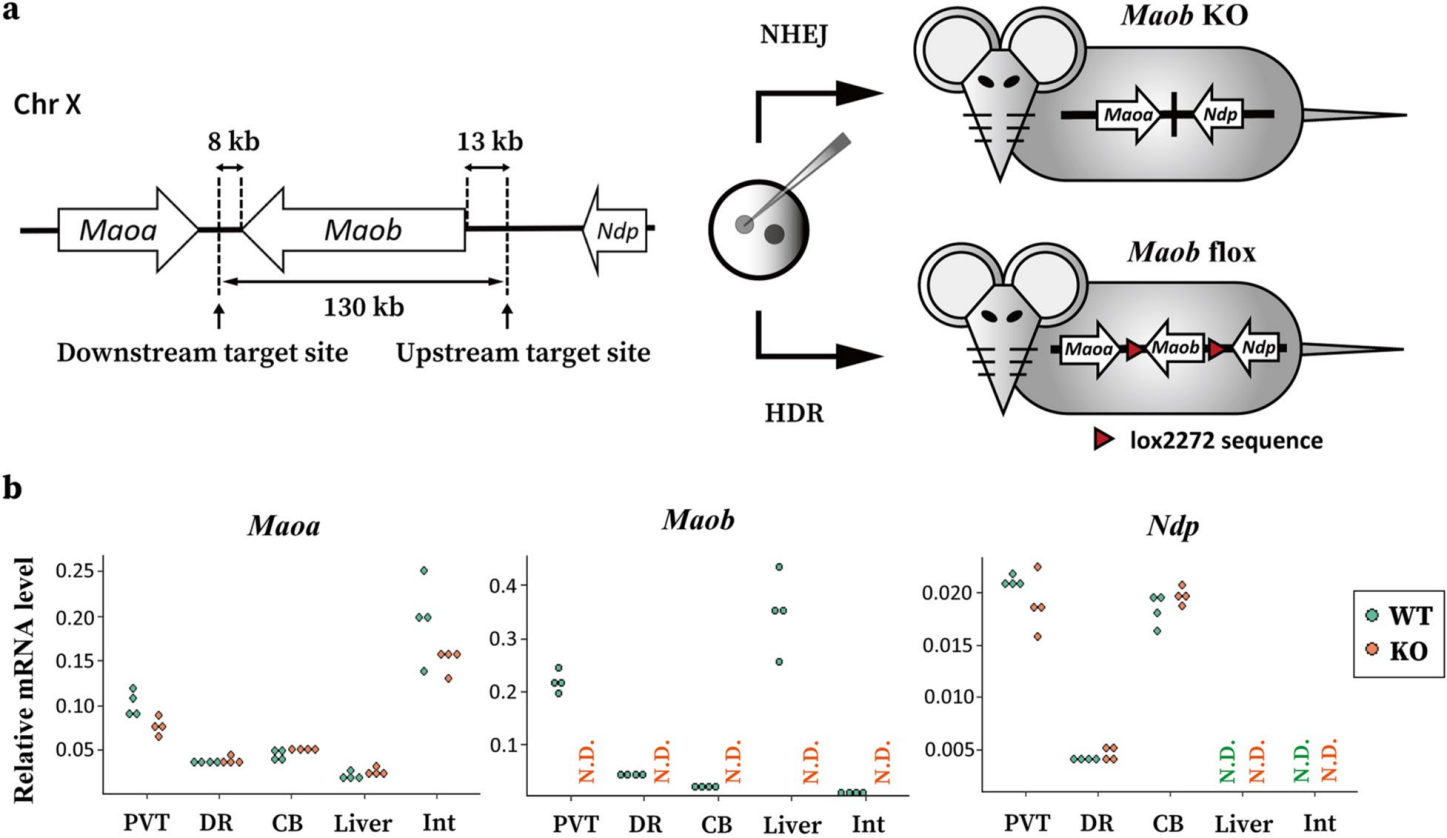
Generation of *Maob* KO mice and *Maob* flox mice. (**a**) A strategy for simultaneous generation of *Maob* KO and *Maob* flox alleles using the CRISPR/Cas9 system. The two gRNA were designed to target the flanking regions of the *Maob* gene located on the X chromosome (Chr X) and ~ 13-kb upstream of the start codon and ~ 8-kb downstream of the stop codon. A CRISPR/Cas9 cocktail containing two single-stranded DNA donor templates with the lox2272 was microinjected into mouse eggs. Non-homologous end-joining (NHEJ) resulted in the deletion of ~ 130 kb (KO allele) or homology-directed repair (HDR) generated the floxed allele (flox). (**b**) Reverse transcription-quantitative PCR analysis of expression of *Maoa*, *Maob*, and *Ndp* in the PVT, the dorsal raphe (DR), cerebellum (CB), liver, and intestine (Int) of wild-type (WT, *Maob*(+/Y)) and *Maob* KO (KO, *Maob*(−/Y)) mice (*n* = 4 for each group). *Maob* expression was not detected in any tissues of *Maob* KO mice. N.D., not detected. mRNA levels of *Maoa* or *Ndp* did not differ significantly between genotypes (Student’s *t-*test with Bonferroni correction).

To develop *Maob* KO and *Maob* flox alleles, we cut the 5ʹ- and 3ʹ-flanking regions of the *Maob* gene in mouse fertilized eggs using the CRISPR/Cas9 system (Fig. 2a). The injected CRISPR/Cas9 cocktail contained two kinds of single-stranded oligonucleotides (ssODNs) comprising the lox2272 sequence and homology arms, which allowed for homology-directed repair to induce insertion of the lox2272 sequence at the two cut positions or non-homologous end-joining of the two cut ends to induce deletion of the *Maob* gene (Supplementary Fig. S1). According to the results of genotyping PCR, we obtained four individuals harboring the *Maob* KO allele and four individuals harboring the *Maob* flox allele. Sanger sequencing confirmed that the *Maob* gene was deleted or floxed with lox2272 (Supplementary Fig. S1). We then performed whole-exome sequencing using one of the third final generation (F3) offspring from *Maob* KO or *Maob* flox lines and verified that they harbored no damaging mutations that might have occurred because of the off-target activity of Cas9.

Reverse transcription-quantitative PCR analysis showed no *Maob* expression in any tissues collected from the global *Maob* KO mice (Fig. 2b). We then determined whether the 130-kb deletion affected the expression of adjacent genes, *Maoa* and *Ndp*. These mRNA levels normalized against either *Actb* (Fig. 2b), *Gapdh*, or *calbindin 2* (*Calb2*; a PVT neuron marker) (Supplementary Fig. S1) did not change significantly in any of the tissues.

### PEA levels in the PVT and other brain regions are elevated in global Maob KO mice

**Figure 3:**
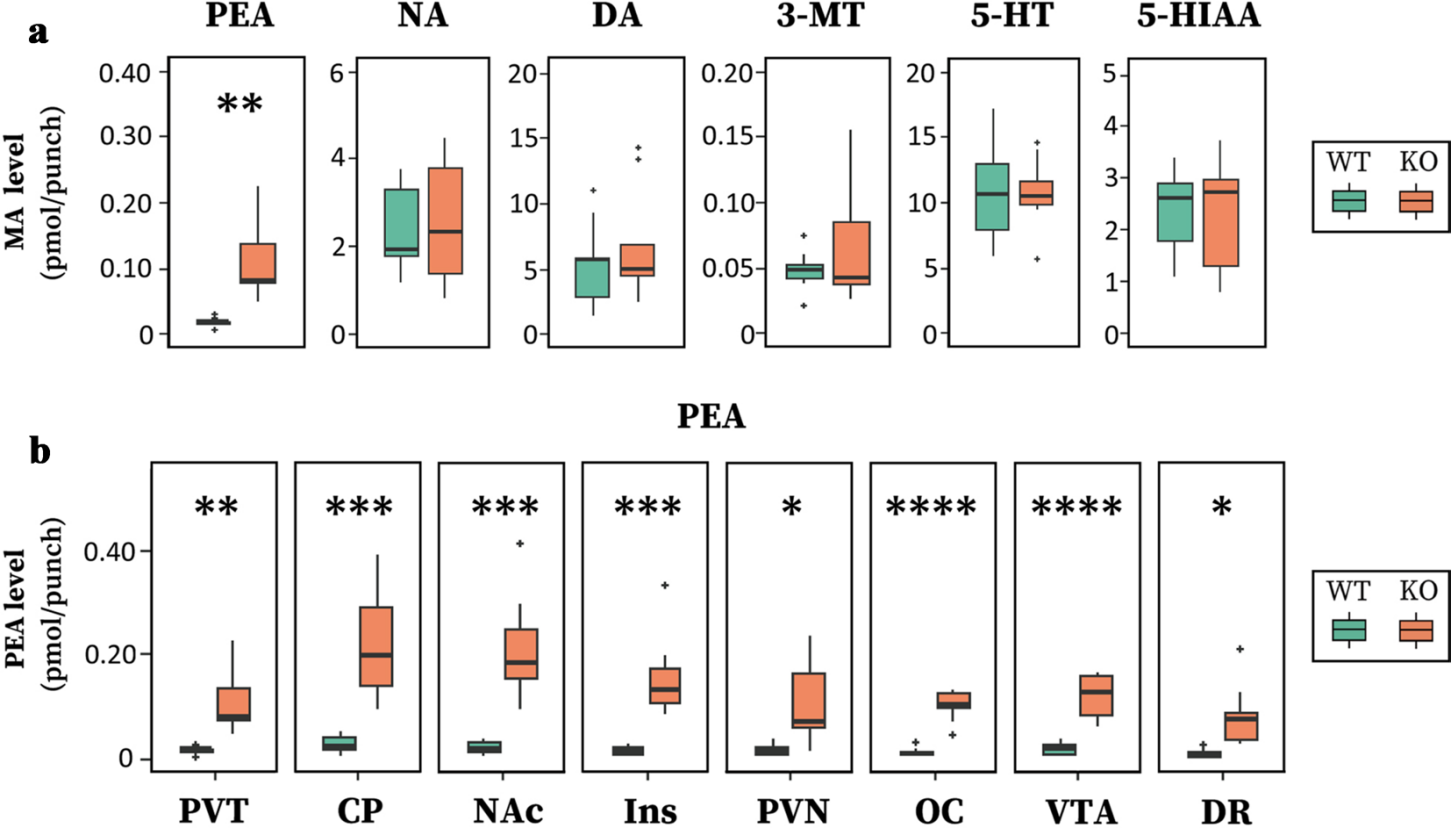
PEA levels are elevated in the PVT and other brain regions in global *Maob* KO mice. (**a**) MAs, trace amines, and their metabolites in the PVT of wild-type (WT, *Maob*(+/Y)) and *Maob* KO (KO, *Maob*(−/Y)) mice (*n* = 9 for each group). The amounts of substances are described as pmol/punch. Boxplots show five statistics: median (horizontal bar), first and third quartiles (lower and upper hinges), and largest and smallest values no further than the 1.5-fold interquartile range (upper and lower whiskers). Data beyond the 1.5-fold interquartile range are plotted individually as outliers. (**b**) PEA levels in the PVT and other brain regions of WT and *Maob* KO mice. Data for PEA levels in the PVT are the same as the PEA levels shown in the (*a*). Two-way ANOVA showed a significant difference depending on the genotype × region interaction (genotype: *df* = 1, *F* = 197.749, *P* < 2 × 10^−16^; region: *df* = 7, *F* = 5.407, *P* = 1.89 × 10^−5^; and genotype × region interaction: *df* = 7, *F* = 3.712, *P* = 0.00109). *P < 0.05, **P < 0.01, ***P < 0.001, ****P < 0.0001, Student’s *t-*test with Bonferroni correction. *CP* caudate-putamen, *NAc* nucleus accumbens, *Ins* insular cortex, *PVN* paraventricular hypothalamic nucleus, *OC* occipital cortex, *VTA* ventral tegmental area, *DR* dorsal raphe nucleus.

Global *Maob* KO mice and their WT littermates were anesthetized, and the brains were fixed with focused microwave irradiation to obtain a snapshot of steady-state substance (MAs and metabolites) levels^43^. We sampled the PVT region (φ1 mm × 2 mm thickness; Supplementary Fig. S2) and measured PEA, NA, DA, 3-methoxytyramine (3-MT), 5-HT, and 5-hydroxyindoleacetic acid (5-HIAA) using a highly sensitive and specific LC–MS/MS method. Representative chromatograms are presented in Supplementary Fig. S3. Two-way analysis of variance (ANOVA) with the main effect of genotype and substance showed a significant difference depending on the genotype × substance interaction (genotype: *df* = 1, *F* = 24.15, *P* = 3 × 10^−6^; substance: *df* = 6, *F* = 39.11, *P* < 2 × 10^−16^; and genotype × substance interaction, *df* = 6, *F* = 22.88, *P* < 2 × 10^−16^). When comparing the genotypes for each substance, only PEA was markedly increased in *Maob* KO mice (*P* = 0.0014, Student’s *t*-test with Bonferroni correction) (Fig. 3a). Seven other brain regions (caudate-putamen, NAc, Ins, paraventricular hypothalamic nucleus, occipital cortex, VTA, and DR) were also sampled and assayed (Supplementary Fig. S2). PEA levels were elevated in the other brain regions, as well as in the PVT (Fig. 3b). No other MAs or metabolites in any brain region differed significantly between *Maob* KO and WT mice (Supplementary Fig. S4).

### Potential alternations of PEA levels in brain-specific Maob KO mice

**Figure 4:**
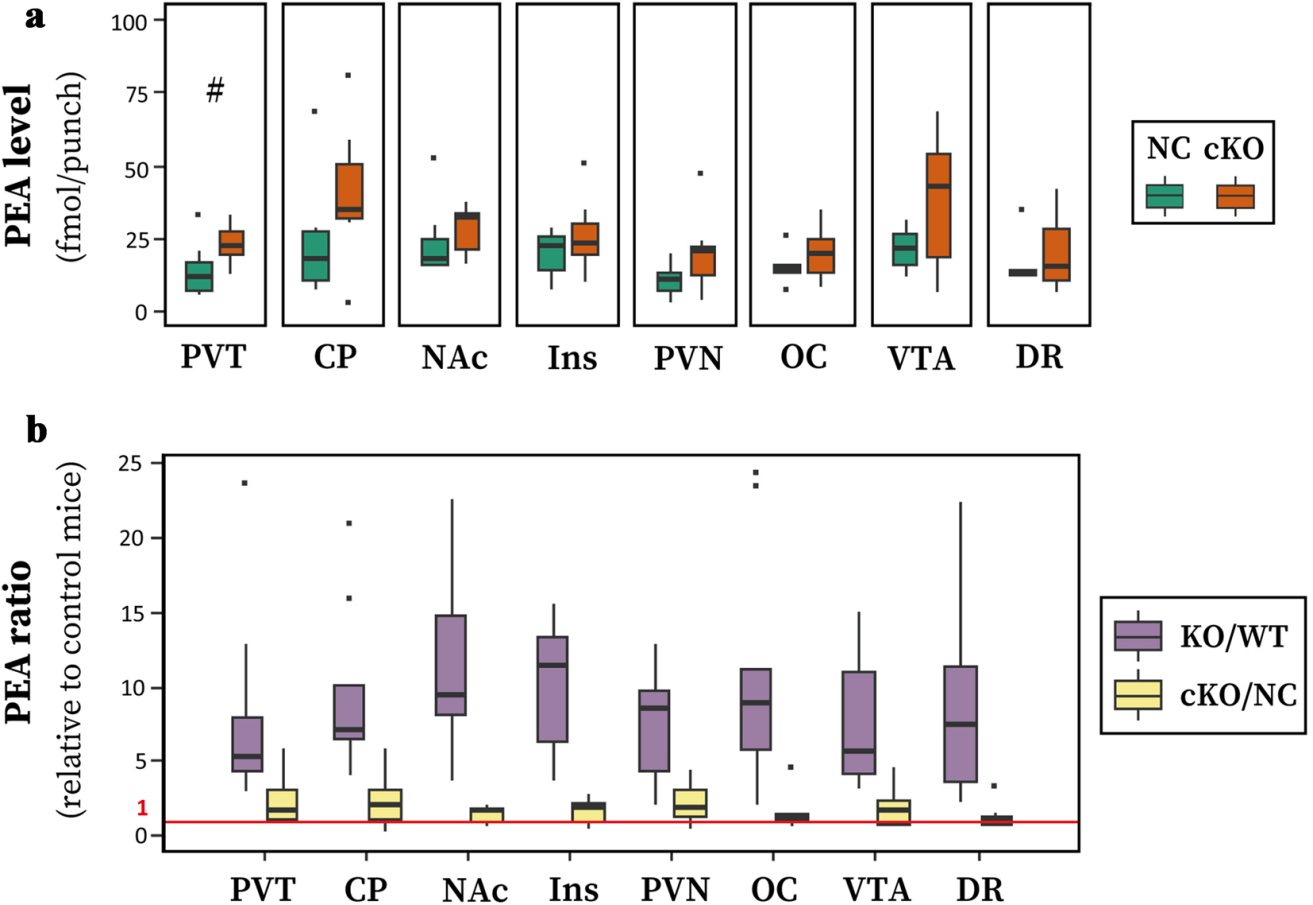
Potential alternation of PEA levels in brain-specific *Maob* KO mice. (**a**) PEA levels in the PVT and other brain regions of control *Nestin*-Cre (NC, *Maob*(+/Y);*Nestin*(Tg/+)) and brain-specific *Maob* KO (cKO, *Maob*(flox/Y);*Nestin*(Tg/+)) mice (*n* = 7 for each group). ^#^*P* = 0.076 by Student’s *t*-test. (**b**) The ratio of PEA levels in *Maob* KO mice to those in control mice. PEA ratios in each region of global *Maob* KO (*Maob*(−/Y)) to wild-type (*Maob*(+/Y)) littermate mice (KO/WT, *n* = 9) and that of cKO (*Maob*(flox/Y);*Nestin*(Tg/+)) to *Nestin*-Cre (*Maob*(+/Y);*Nestin*(Tg/+)) littermate mice (cKO/NC, *n* = 7) are shown. The red horizontal line indicates a ratio of 1. Two-way ANOVA showed a significant difference depending on the genotype (genotype: *df* = 1, *F* = 86.327, *P* = 1.43 × 10^−15^; region: *df* = 7, *F* = 0.426, *P* = 0.884; and genotype × region interaction: *df* = 7, *F* = 0.597, *P* = 0.757).

We then examined brain-specific *Maob* KO (that is, *Maob*(flox/Y);*Nestin*-Cre), in which MAO-B enzymes in the peripheral tissues remained intact. We measured the substances in the PVT and other brain regions, paying particular attention to PEA. We found that the PEA level was also elevated in brain-specific *Maob* KO mice (Fig. 4a), but the extent of elevation was less than that in global *Maob* KO mice (Fig. 4b). Two-way ANOVA with the main effect of genotype and region showed a significant difference depending on the genotype, but not on the genotype × region interaction (genotype: *df* = 1, *F* = 11.595, *P* = 0.000967; region: *df* = 7, *F* = 2.956, *P* = 0.00755; and genotype × region interaction: *df* = 7, *F* = 0.471, *P* = 0.853). In the PVT, Student’s *t-*test showed that PEA levels tended to increase (*P* = 0.076) (Fig. 4a). Comparison of the ratio of PEA increase in brain-specific KO mice to control mice relative to that of global KO mice to WT mice revealed a significantly smaller ratio in brain-specific KO mice (Fig. 4b). The other MAs and metabolites were not significantly altered in brain-specific KO mice (Supplementary Fig. S5).

### Plasma PEA level is elevated in global Maob KO mice but not in brain-specific Maob KO mice

**Figure 5:**
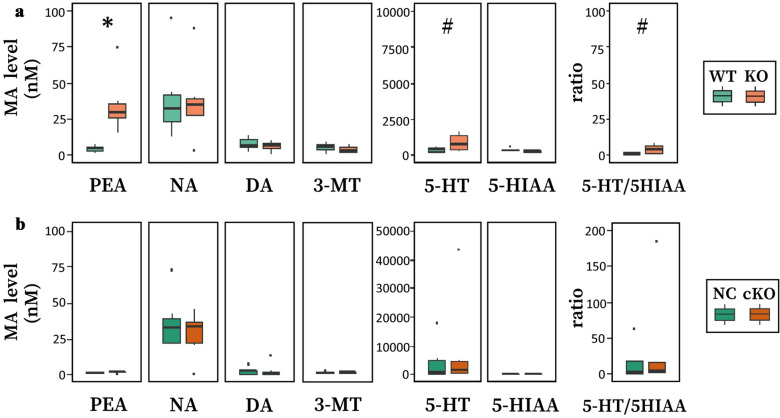
Plasma PEA levels are elevated in global KO mice but not in brain-specific cKO mice. (**a**) Plasma concentrations in wild-type (WT, *Maob*(+/Y)) and *Maob* KO (KO, *Maob*(−/Y)) mice (*n* = 6 for each group). Two-way ANOVA showed a significant difference depending on the genotype × substance interaction (genotype: *df* = 1, *F* = 3.006, *P* = 0.0874; substance: *df* = 6, *F* = 13.650, *P* = 3.4 × 10^−10^; and genotype × substance interaction: *df* = 6, *F* = 2.686, *P* = 0.0210). *P < 0.05, Student’s *t-*test with Bonferroni correction. (**b**) Plasma concentrations in control *Nestin*-Cre (NC, *Maob*(+/Y);*Nestin* (Tg/+)) and brain-specific *Maob* KO (cKO, *Maob*(flox/Y);*Nestin*(Tg/+)) mice (*n* = 7 for each group). Two-way ANOVA showed no significant difference depending on the genotype × substance interaction (genotype: *df* = 1, *F* = 0.305, *P* = 0.582; substance: *df* = 6, *F* = 4.642, *P* = 0.00040; and genotype × substance interaction: *df* = 6, *F* = 0.440, *P* = 0.850). ^#^*P* < 0.07, Student’s *t*-test.

PEA passes across the BBB, and plasma PEA is distributed in the brain parenchyma^39,41^. The observed larger increase in PEA level in the PVT of global *Maob* KO mice could be attributed to increased plasma PEA levels induced by peripheral MAO-B deficiency. To test this possibility, we measured plasma PEA levels, as well as those of other MAs and metabolites, in global KO mice and brain-specific KO mice. Plasma PEA level was higher in global KO mice than in WT mice (*P* = 0.0325) (Fig. 5a), whereas plasma PEA level in brain-specific KO mice was not significantly increased relative to that in control *Nestin*-Cre mice (Fig. 5b). Additionally, the 5-HT concentration and 5-HT/5-HIAA ratio tended to increase in the plasma of global KO mice. These results suggest that PEA is metabolized by MAO-B in peripheral tissues, and that elevated levels of plasma PEA induced increases in PEA levels in brains of global *Maob* KO mice.

## Discussion

MA neurotransmission profoundly affects a wide range of brain areas and nuclei, the functions of which are highly differentiated and interdependent. However, it is challenging to examine the MA metabolism of small nuclei, such as the PVT, whose volume is at most 1 mm^3^ in mice. A recent study using imaging MS detected classical MAs at relatively high abundance in the PVT^24^, although PEA was not investigated. In the present study, we successfully measured trace amine PEA levels in small brain regions, including the PVT, using a microwave fixation method and high-sensitivity LC–MS/MS. Comparison of PEA levels in the PVT of WT, global *Maob* KO, and brain-specific *Maob* KO mice (Figs. 3, 4) revealed that PEA is a substrate of MAO-B in the PVT.

In global *Maob* KO mice, blood PEA levels were significantly increased (Fig. 5), likely due to the suppression of PEA degradation in peripheral tissues. Because PEA passes through the BBB^39–42^, the increase in PEA levels in the brains of global KO mice is the sum of the local suppression of PEA degradation and the influx of PEA from the periphery. However, the influence of the former is likely insignificant, because we did not detect any increase in PEA levels in brain tissues, except for the PVT, from brain-specific *Maob* KO mice (Fig. 4). This study illustrates the need for careful interpretation of PEA levels in the brains of global *Maob* KO mice or following systemic *Maob* inhibitor treatment.

A recent study reported that noradrenergic afferent terminals of the locus coeruleus to PVT projection neurons mediate DA release in the PVT^17^, which leaves open the possibility that NA and DA could also be MAO-B substrates in the PVT. Indeed, MAO-B is co-expressed in astrocytes along with vesicular monoamine transporter 2 (VMAT2) and organic cation transporter 3 and could metabolize DA under conditions of VMAT2 dysfunction^9^. Additionally, DA is also converted to 3-MT (Fig. 1), which could be another candidate MAO-B substrate. Moreover, 5-HT levels in the brains of *Maoa*/*Maob* double KO mice were higher than those of single *Maoa* KO mice, suggesting that MAO-B partly contributes to 5-HT metabolism^44^. However, the present study did not identify the metabolisms of these MAs by MAO-B in the PVT (Fig. 3a and Supplementary Fig. S5). Furthermore, the lack of DA alteration in *Maob* KO mice is consistent with the findings that DA is metabolized predominantly by MAO-A rather than MAO-B in rodents^1,45–48^. Analysis of metabolites is important for investigating NA and DA metabolism, however, these concentrations in the PVT, which is a small region, were not determined in this study.

This study has several limitations. First, the genetic KO system, either global or brain-specific, deleted the *Maob* gene from the immediate-early stage of ontogeny. There is a possibility that unidentified compensatory mechanisms for metabolic homeostasis might occur and influence MA levels. Second, our measurements of the MAs and metabolites were performed at steady state. We could not evaluate the effect of MAO-B deficit on MA dynamics after stimulation, task, or stress, each of which can accelerate MA release. Third, this investigation was a target analysis of the selected MAs and metabolites. We cannot exclude the possibility that MAO-B can metabolize unidentified substrates. In particular, it should be noted that there are many trace amines in the brain other than PEA, including tyramine, tryptamine, and octopamine, which are reportedly common substrates of both MAO types according to biochemical studies^3^. Additionally, we have to consider also the fact that MAO-B plays a role in the putrescine-degradation pathway, resulting in the production of gamma-aminobutyric acids in glial cells^8,10^. Another limitation of the study is that the metabolism of DA, NA, and PEA was evaluated solely by measuring substrate concentrations. The PVT is so small that it was necessary to measure many substrates simultaneously in one measurement. However, metabolites produced from DA (i.e., 3,4-dihydroxyphenylacetic acid [DOPAC], and homovanillic acid), NA (i.e., 3-methoxy-4-hydroxyphenylglycol, and vanillylmandelic acid), and PEA (i.e., phenylacetic acid) could not be quantified because of the low ionization efficiency and the use of microwave irradiation for brain-sample preparation, which avoided postmortem degradation and maintained high MA concentrations and low metabolite concentrations^43^. A study using bulk tissues showed that the ratio of DOPAC to DA prepared by microwave irradiation was ~ sevenfold lower than that prepared by conventional method^43^. Because the PVT is innervated predominantly by serotonergic neurons rather than dopaminergic neurons^12,21^ and DA is metabolized primarily by MAO-A in rodents^1,45–48^, we focused on PEA and 5-HT metabolism in this study.

In summary, these findings revealed a potential physiological role of MAO-B in the PVT. The PVT is anatomically in contact with the third ventricle (3 V) and positioned close to the choroid plexus at the 3 V, making it a potential entry point for PEA from the peripheral blood. PEA is known as “the body’s own amphetamine” since it has pharmacological effects similar to those of amphetamine, especially its effect on altering MA transporter function^29,34,49–51^. This action potentially disturbs the fine-tuning of MA neurotransmission in the PVT, considering that many MA receptors are localized in this region^11,17,22,23^. If MAO-B plays a role in protecting neurons receiving MA neurotransmission from perturbations by PEA as a “false transmitter”^4,7^, it is reasonable that MAO-B is highly expressed in the PVT. However, PEA primarily demonstrates a high affinity for trace amine-associated receptor 1 (TAAR1), which is a G-protein coupled receptor identified in 2001^52–54^. *Taar1* mRNA is expressed in monoaminergic nuclei such as the VTA and DR^52,55^, and TAAR1 activation can modulate monoaminergic neural activity and animal behavior^54–57^. The mechanism of action of TAAR1 is intriguing from a clinical standpoint, because a recent study reported that a TAAR1 agonist showed significant therapeutic effects in patients with schizophrenia^58^. In this case, PEA could be a valuable neuromodulator that reflects external and internal environments, such as diet and gut microbiota, and MAO-B could be a regulator of this PEA signaling. Here, we described the potential role of MAO-B in the PVT, with a focus on PEA metabolism; however, there are many other MAs in the brain that could be metabolized by MAO-B. In future studies, further investigation will be needed for a more comprehensive metabolome analysis of PVT-specific and cell type-specific MAO-B inactivation. This will enable elucidation of the physiological importance of MAO-B in the PVT, as a promising target region for understanding the mechanism of mood regulation and emotional behaviors underlying mental disorders.

## Materials and methods

All animal care and experimental procedures were approved by the RIKEN Wako Animal Experiment Committee (approval number: W2019-2-040[4]). All animal experiments were performed in accordance with ARRIVE guidelines and the guidelines for the proper conduct of animal experiments published by the Science Council of Japan.

### Animals

The experiments were performed using adult male mice, with all animals housed with ad libitum access to food and water. The animal care unit was maintained on a 12-:12-h light–dark cycle (08:00–20:00) with controlled temperature (23 ± 2 °C) and humidity (50 ± 15%). *Maob* KO mice and *Maob* flox mice were developed using the CRISPR/Cas9 system on the background of the C57BL/6J strain. Male C57BL/6J WT mice (C57BL/6JJcl; CLEA Japan, Tokyo, Japan) were crossbred with female *Maob*(−/+) mice to obtain male *Maob*(−/Y) mice and their littermate WT (*Maob*(+/Y)) mice as controls. A *Nestin*-Cre transgenic mouse line (B6.Cg^Tg(Nes-cre)1kln/J^) was obtained from the Jackson Laboratory (Bar Harbor, ME, USA). Male *Nestin*-Cre(Tg/Tg) mice were crossbred with female *Maob*(flox/+) mice to obtain male *Maob*(flox/Y);*Nestin*(Tg/+) mice, with their littermate *Maob*(+/Y);*Nestin*(Tg/+) mice used as controls.

### Generation of Maob KO mice and Maob flox mice using the CRISPR/Cas9 system

We cut the two target positions using the CRISPR/Cas9 system and generated a large deletion at the *Maob* locus by non-homologous end-joining or inserted the lox2272 sequences into the two cut positions by homology-directed repair. For this purpose, we designed two crRNAs targeting the upstream and downstream regions of the *Maob* gene and two ssODNs containing the lox2272 sequences with 80-nt homology arms (Supplementary Fig. S1). We prepared a CRISPR/Cas9 cocktail containing 100 ng/μL Cas9 protein (Alt-R S.p. Cas9 Nuclease V3), 10 μM tracrRNA (Alt-R CRISPR-Cas9 tracrRNA), 5 μM each crRNA (Alt-R CRISPR-Cas9 crRNA), 10 ng/μL of each ssODN (PAGE Ultramer DNA Oligo) in nuclease-free duplex buffer with 100 mM potassium acetate and 30 mM 4-(2-hydroxyethyl)-1-piperazine ethanesulfonic acid (HEPES). These constructs for the CRISPR/Cas9 system were purchased from Integrated DNA Technologies (Coralville, IA, USA). The cocktail was injected into the pronuclei of 500 fertilized mouse eggs. A total of 286 embryos were transplanted to surrogate mothers, and 53 pups were obtained (F0 generation). We performed genotyping by PCR using genomic DNA extracted from tail biopsies using the following primer sequences: to detect the *Maob* KO allele, AGAGCAAGGCCACTCTCAAAG and AGAGGAAGCTGAGACTCTGACTG; to detect lox2272 insertion in the upstream region, AGAGCAAGGCCACTCTCAAAG and CTAACAGTGGCCTCAAAGCAG; and to detect lox2272 insertion in the downstream region, CATCCATGTGCTAAACAATGC and AGAGGAAGCTGAGACTCTGACTG. F0 mice carrying the *Maob* KO allele (four individuals) or *Maob* flox allele (four individuals) were crossbred with C57BL/6J WT mice. Sanger sequencing was performed to confirm the large deletion at the *Maob* locus or correct insertion of the lox2272 sequences in the F1 offspring.

### Assessment of off-target mutations by whole-exome sequencing

We performed whole-exome sequencing using DNA samples from the F3 offspring of *Maob* KO and *Maob* flox lines for comparison with a C57BL/6J WT mouse. Genomic DNA was extracted from the kidneys using the GenElute mammalian genomic DNA miniprep kit (Sigma-Aldrich, St. Louis, MO, USA). DNA capture, library preparation, DNA sequencing, and variant calling were performed by Riken Genesis (Tokyo, Japan). Briefly, a DNA library was prepared following the SureSelect Target Enrichment System workflow using a SureSelect Mouse All Exon kit (Agilent Technologies, Santa Clara, CA, USA). Sequencing was performed on a NovaSeq 6000 platform (Illumina, San Diego, CA, USA) to produce 150-bp reads. Sequencing reads were aligned to the mouse reference (mm10) the Burrows–Wheeler Aligner (v.0.7.10; http://bio-bwa.sourceforge.net/). Annotation for all variants was made using dbSNP151, CCDS (NCBI, release 21), and RefSeq (UCSC Genome Browser, dumped 20181125). We applied a further filtering process (Supplementary Table S1) to identify the private variants carried by each F3 mouse. The *Maob* KO mouse (female, *Maob*(−/+)) harbored a frameshift mutation (Chr 16: 23114138, ins A) in exon 10 of *Rfc4* gene. Because the probability of a loss-of-function intolerant (pLi) score of the *Rfc4* gene was 0 according to gnomAD (https://gnomad.broadinstitute.org/), we judged that this frameshift mutation in a heterozygous manner could not affect the results of the following experiments. No private variants were detected in the *Maob* flox mouse (male, *Maob*(flox/Y)). We performed the following experiments using F4 and F5 mice.

### Validation of loss of Maob expression by reverse transcription-quantitative PCR

We assayed the mRNA expression of *Maoa*, *Maob*, *Ndp*, and *Calb2* using male *Maob*(−/Y) mice and their littermate *Maob*(+/Y) (12–13 weeks old, *n* = 4 for each group). *Actb* and *Gapdh* were also measured as internal normalizers. We collected the PVT, DR, cerebellum, liver, and intestine from decapitated individuals. Total RNA was isolated from the tissues using TRIzol reagent (Thermo Fisher Scientific, Waltham, MA, USA) and extracted using the RNeasy mini kit (Qiagen, Venlo, Netherlands) according to the manufacturer instructions. The RNA integrity number was measured using an Agilent 2100 bioanalyzer (Agilent Technologies) and confirmed to be in the range of 7.3 to 9.6. RNA was then reverse transcribed using SuperScript III First-Strand Synthesis SuperMix (Thermo Fisher Scientific). Target mRNA levels were analyzed using specific primers (Supplementary Table S2) and TB Green Premix Ex Taq II (Takara Bio, Shiga, Japan). Quantitative PCR was performed using a QuantStudio 12 K Flex (Thermo Fisher Scientific). All PCRs were performed in quadruplicate. When the threshold cycle (Ct) value was > 30 in more than two PCR reactions, mRNA expression was evaluated as being ’not detected’.

### Analysis of MAs and metabolites by LC–MS/MS

Animals aged 15 to 30 weeks were used for LC–MS/MS experiments. The chemicals and reagents were purchased as follows: 2-phenylethylamine hydrochloride was obtained from Tokyo Chemical Industry (Tokyo, Japan). l-(−)-norepinephrine (+)-bitartrate salt monohydrate, dopamine hydrochloride, 5-hydroxytryptamine creatinine sulfate monohydrate, 3-methoxytyramine hydrochloride, 5-hydroxyindole-3-acetic acid, and 3,4-dihydroxybenzylamine hydrobromide were obtained from Sigma-Aldrich. Dihydroxybenzylamine (DHBA) was used as an internal standard. LC–MS-grade formic acid (FA) and acetonitrile (ACN) were purchased from Fujifilm Wako Pure Chemical Corporation (Osaka, Japan). Ascorbic acid (AA) was purchased from Tokyo Chemical Industry.

#### Brain sample preparation for LC–MS/MS

Animals were anesthetized with isoflurane and subjected to focused microwave irradiation (5 kW, 1.05 s) using an MMW-05 apparatus (Muromachi Kikai, Tokyo, Japan). This was performed between 13:00 and 15:00 h. After microwave irradiation, brains were collected and sliced using a brain matrix (2-mm thickness for the PVT and paraventricular hypothalamic nucleus; 1-mm thickness for bilateral sampling of the other regions). The seven regions were set as controls; the NAc, Ins, and DR were anatomically connected to the PVT and the other regions (the caudate-putamen, paraventricular hypothalamic nucleus, occipital cortex, and VTA) were not connected to the PVT. Each target region was collected by punch biopsy (φ1.0 mm) on ice (Supplementary Fig. S2). Punch biopsies were purchased from Kai Industries (Gifu, Japan), and the punched specimens were stored at − 80 °C and used for subsequent extraction treatment within one week.

Extraction of MAs and metabolites was performed using a modified method based on a previous report^59^. We added 90 μL of 1.89% FA/1 mM AA in ice-cold water to each sample, which was then homogenized using an ultrasonic probe (VCX 130; Sonics and Materials, Newtown, CT, USA) at 20% power for 20 s. We then added 10 μL of 1 μM DHBA/1 mM AA in ice-cold water as an internal control, and 10 μL of 1 mM AA in ice-cold water was added as a replacement for addition of the standard substance for calibration curve preparation. After centrifugation at 14,000×*g* for 15 min at 4 °C, 85 μL of the supernatant was collected, and 340 μL of ACN with 1% FA/1 mM AA was added for deproteinization. After centrifugation at 14,000×*g* for 5 min at 4 °C, 400 μL of the supernatant was collected, evaporated using a concentrator (CVE-3100 and UT-2000, Tokyo Rikakikai, Tokyo, Japan), and stored at − 80 °C until the LC–MS/MS assay.

For quantitative analysis, we generated calibration curves for the target MAs and metabolites in each batch experiment. In advance, we applied microwave irradiation to a C57BL/6J WT mouse, harvested the whole brain, prepared the homogenate within 90 μL of 1.89% FA/1 mM AA in ice-cold water per mg tissue weight, and stored the samples at − 80 °C until use. Five aliquots (90 μL) of the homogenate were taken, and we added with 10 μL of 1 μM DHBA/1 mM AA in ice-cold water as an internal control and 10 μL of standard substance solution with 1 mM AA to each aliquot. The standard substance solutions included mixtures of PEA (0–50 μM [0, 0.78125, 3.125, 12.5, 50 μM]), NA (0–5000 μM), DA (0–5000 μM), 3-MT (0–50 μM), 5-HT (0–5000 μM), and 5-HIAA (0–5000 μM). We then proceeded with the same extraction treatment used for the brain samples.

#### Preparation of plasma samples for LC–MS/MS analysis

We used animals distinct from the individuals used for the brain LC–MS/MS experiments. The animals were anesthetized with isoflurane, and blood was collected from the heart using needle aspiration. The sampling syringes were rinsed with 0.5 M ethylenediaminetetraacetic acid disodium salt in advance. The blood was centrifuged at 1000×*g* for 20 min at 4 °C, and plasma was collected. We added 10 μL of 1 μM DHBA/1 mM AA in ice-cold water and 10 μL of a standard substance solution along with 1 mM AA to 10 μL of plasma. The standard substance solutions were mixtures of PEA (0–250 nM [0, 3.90625, 15.625, 62.5, and 250 nM]), NA (0–250 nM), DA (0–250 nM), 3-MT (0–250), 5-HT (0–10,000), and 5-HIAA (0–5000 nM). We then added 60 μL of ACN with 1% FA/1 mM AA, followed by centrifugation at 19,000×*g* for 10 min at 4 °C. Finally, 80 μL of the supernatant was collected, evaporated to dryness, and stored at − 80 °C. Quantification was performed using the standard addition method. When the calculated value of the substance levels based on the calibration curve was < 0, the concentration was set to 0 nM (see Supplementary Information).

#### Detection of MAs and metabolites by LC–MS/MS analysis

All dried samples were reconstituted in 30 μL of 1% ACN with 0.1% FA/1 mM AA. After centrifugation, 5 μL of the supernatant was injected into the LC–MS/MS system consisting of a Vanquish UHPLC and TSQ Vantage EMR (Thermo Fisher Scientific). Separation of the analytes was carried out on a YMC Triart C18 column (2.0 × 100 mm). Mobile phases A (0.1% FA) and B (ACN) were used for linear gradient elution: 0–1 min, 0% B; 1–4 min, 0–60% B; 4–4.1 min, 60–95% B; 4.1–6 min, 95% B; 6–6.1 min, 95–0% B; and 6.1–8 min, 0% B. The flow rate was set at 0.3 mL/min. The electrospray conditions were as follows: spray voltage, 3000 V; vaporizer temperature, 450 °C; sheath gas pressure, 50; auxiliary gas pressure, 15; and collision gas pressure, 1.0 mTorr. Argon was used as the collision gas. The mass spectrometer was operated in positive/negative-ion multiple-reaction monitoring (MRM) mode for the detection of the molecular transition of each analyte (Supplementary Table S3). TraceFindersoftware (v.4.0; Thermo Fisher Scientific) was used for data processing. Ion peak detection and peak area integration were performed based on the analysis parameter settings shown in Supplementary Table S4. When the retention time was shifted and the ion peak was detected inadequately by the parameter settings, the ion peak detection and peak area integration were corrected manually.

### Statistical analysis

Data were analyzed using Kyplot (KyensLab, Tokyo, Japan) or R (https://www.r-project.org/). For analysis of reverse transcription-quantitative PCR in the comparison of global *Maob* KO mice with WT mice, Student’s *t*-test with Bonferroni correction was used. For analysis of LC–MS/MS experiments in the comparison of global *Maob* KO mice with WT mice or between brain-specific *Maob* KO mice and control *Nestin*-Cre mice, two-way ANOVA with the main effect of genotype and substance was applied in the PVT and each other respective brain region. When analyzing the PEA levels, a two-way ANOVA with the main effect of genotype and region was applied. Student’s *t-*test was also used in the analyses of LC–MS/MS experiments.

## Supplementary Information

Supplementary Information 1.

Supplementary Information 2.

## Data Availability

All data generated or analyzed during this study are included in this published article (and its Supplementary Information).
