# Supplementary material for: Phenethylamine is a substrate of monoamine oxidase B in the paraventricular thalamic nucleus
Source: Sci Rep. 2022 Jan 7;12:17. doi: 10.1038/s41598-021-03885-6 (PMC8742005; doi:10.1038/s41598-021-03885-6)
Supplement: Supplementary file 2 — Supplementary Information 2. [file 41598_2021_3885_MOESM2_ESM.pdf]

Supplementary information

Phenethylamine is a substrate of monoamine oxidase B in the paraventricular thalamic nucleus

Youhei Obata, Mie Kubota-Sakashita, Takaoki Kasahara*, Masafumi Mizuno, Takahiro Nemoto, and Tadafumi Kato*

*E-mail: takaoki.kasahara@riken.jp; tadafumi.kato@juntendo.ac.jp

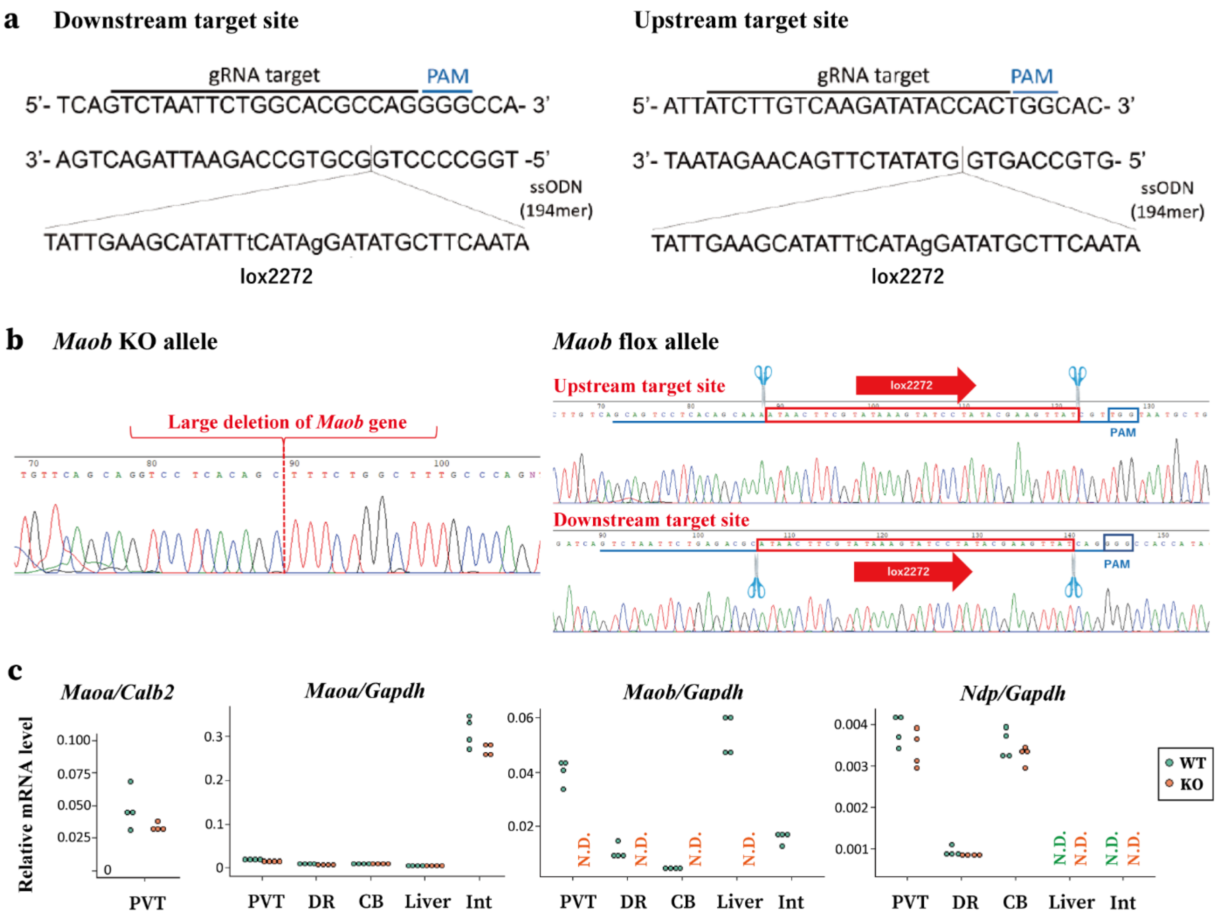

Supplementary Fig. S1: Generation of *Maob* KO mice and *Maob* flox mice. (**a**) Two gRNA target sequences and insertion positions of the lox2272 sequences. PAM, protospacer adjacent motif; ssODN, single-strand oligonucleotide. (**b**) Electropherograms of *Maob* KO and *Maob* flox mice. A vertical dashed line shows the breakpoint of the large deletion at the *Maob* locus (left). Blue underlines show gRNA target sequences, scissor marks show the cut positions by Cas9, and red rectangles show the lox2272 insertions (right). (**c**) Reverse transcription-quantitative PCR analysis of *Maob* KO mice normalized with *Gapdh* or *calbindin 2* (*Calb2*).

The *Maoa* expression in the paraventricular thalamic nucelus (PVT) of wild-type (WT, *Maob*(+/Y)) and *Maob* KO (KO, *Maob*(−/Y)) mice normalized against *Calb2* expression showed no significant difference according to Student's *t*-test. *Maob* expression in the PVT, the dorsal raphe (DR), cerebellum (CB), liver, and intestine (Int) were not detected in *Maob* KO mice. mRNA levels of *Maoa* and *Ndp* normalized against *Gapdh* did not differ significantly between wild-type mice and *Maob* KO mice according to Student's *t*-test with Bonferroni correction. Mice were 12 to 13 weeks old (n = 4 for each group).

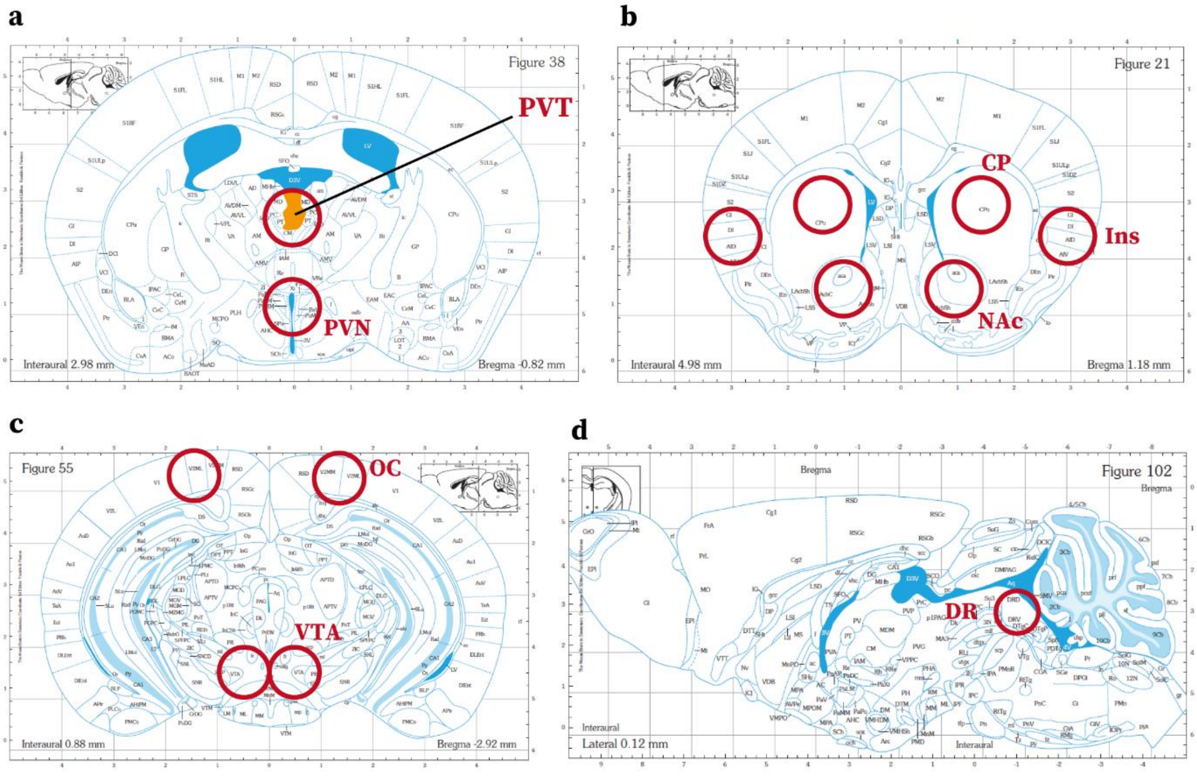

Supplementary Fig. S2: Schematic locations of punch biopsies of the paraventricular thalamic nucleus (PVT) and other brain regions. *The Mouse Brain in Stereotaxic Coordinate, Compact (3rd Edition)* was used as a reference for the punch biopsies of the PVT and other brain regions. The red circles show the locations of biopsy punches (φ1.0 mm). (**a**) The anterior side of a coronal slice (2-mm thickness) containing the PVT and the paraventricular hypothalamic nucelus (PVN). The orange area shows the PVT. (**b**) A coronal slice (1-mm thickness) containing the caudate-putamen (CP), nucleus accumbens (NAc), and insular cortex (Ins). (**c**) A coronal slice (1-mm thickness) containing the occipital cortex (OC) and ventral

tegmental area (VTA). (**d**) The dorsal raphe nucleus (DR) was collected from a sagittal slice (1-mm thickness). The CP, NAc, Ins, OC, VTA, and DR were sampled bilaterally and combined into one sample for each region.

WT

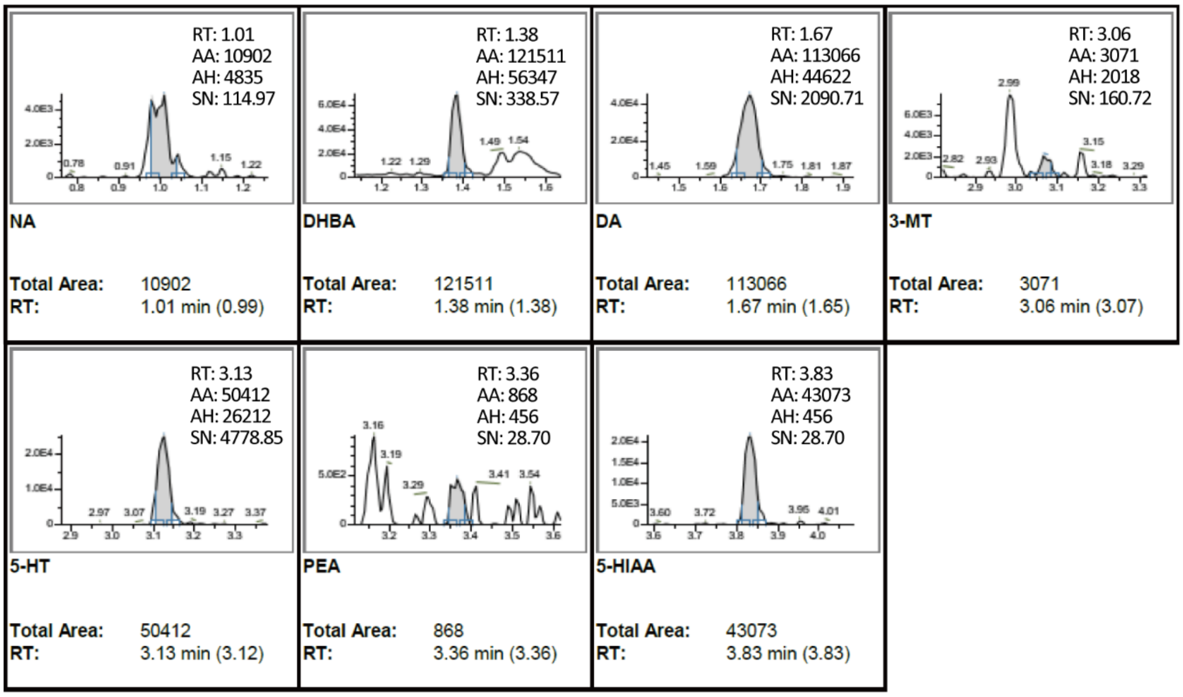
RT: 1.01
AA: 10902
AH: 4835
SN: 114.97
NA
Total Area: 10902
RT: 1.01 min (0.99)
RT: 1.38
AA: 121511
AH: 56347
SN: 338.57
DHBA
Total Area: 121511
RT: 1.38 min (1.38)
RT: 1.67
AA: 113066
AH: 44622
SN: 2090.71
DA
Total Area: 113066
RT: 1.67 min (1.65)
RT: 3.06
AA: 3071
AH: 2018
SN: 160.72
3-MT
Total Area: 3071
RT: 3.06 min (3.07)
RT: 3.13
AA: 50412
AH: 26212
SN: 4778.85
5-HT
Total Area: 50412
RT: 3.13 min (3.12)
RT: 3.36
AA: 868
AH: 456
SN: 28.70
PEA
Total Area: 868
RT: 3.36 min (3.36)
RT: 3.83
AA: 43073
AH: 456
SN: 28.70
5-HIAA
Total Area: 43073
RT: 3.83 min (3.83)

***Maob* KO**

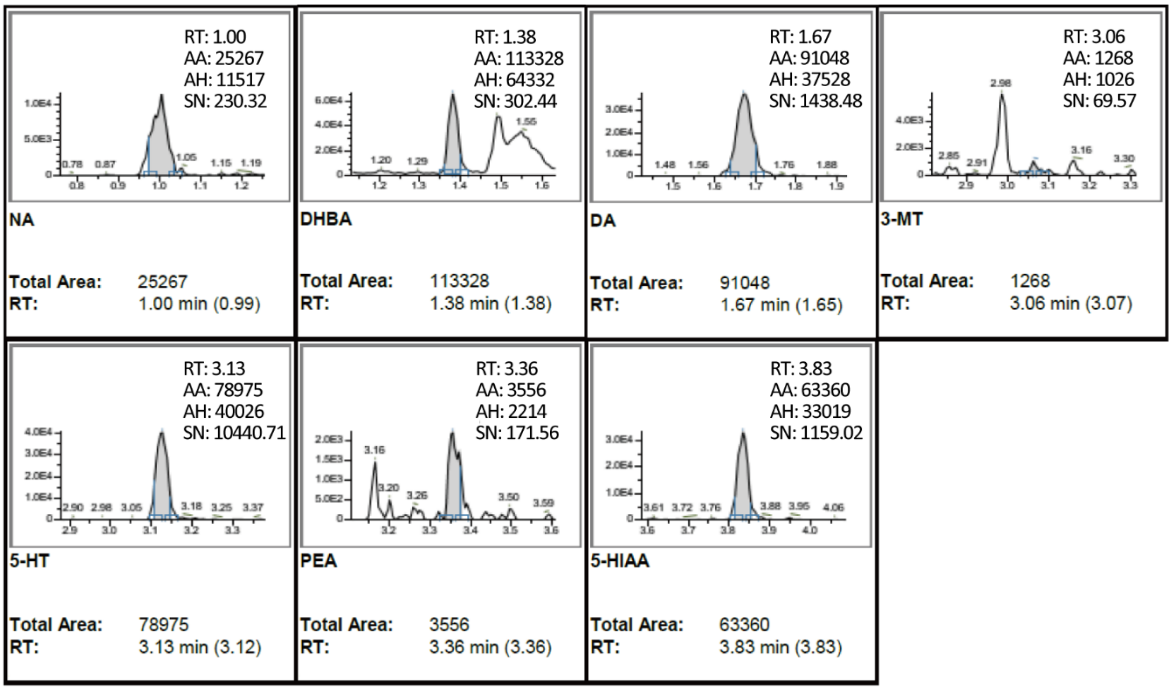
RT: 1.00
AA: 25267
AH: 11517
SN: 230.32
NA
Total Area: 25267
RT: 1.00 min (0.99)
RT: 1.38
AA: 113328
AH: 64332
SN: 302.44
DHBA
Total Area: 113328
RT: 1.38 min (1.38)
RT: 1.67
AA: 91048
AH: 37528
SN: 1438.48
DA
Total Area: 91048
RT: 1.67 min (1.65)
RT: 3.06
AA: 1268
AH: 1026
SN: 69.57
3-MT
Total Area: 1268
RT: 3.06 min (3.07)
RT: 3.13
AA: 78975
AH: 40026
SN: 10440.71
5-HT
Total Area: 78975
RT: 3.13 min (3.12)
RT: 3.36
AA: 3556
AH: 2214
SN: 171.56
PEA
Total Area: 3556
RT: 3.36 min (3.36)
RT: 3.83
AA: 63360
AH: 33019
SN: 1159.02
5-HIAA
Total Area: 63360
RT: 3.83 min (3.83)

Supplementary Fig. S3: Representative LC-MS/MS chromatograms of MAs and metabolites in punch biopsies of the PVT. The upper seven panels show chromatograms for a wild-type (WT, *Maob*(+/Y)) mouse and the lower panels show those for a global *Maob* KO (KO, *Maob*(−/Y)) mouse. Data processing was performed using TraceFinder software (v.4.0; Thermo Fisher Scientific). RT, retention time; AA, peak area; AH, peak height; SN, signal-noise ratio. The setpoints of RT for multiple reaction monitoring of each substance are shown in parentheses. The gray areas are integrated for AA or total area. DA, dopamine; DHBA, dihydroxybenzylamine; 5-HT, 5-hydroxytryptamine; 5-HIAA, 5-hydroxyindole acetic acid; NA, noradrenaline; 3-MT, 3-methoxytyramine; PEA, phenethylamine.

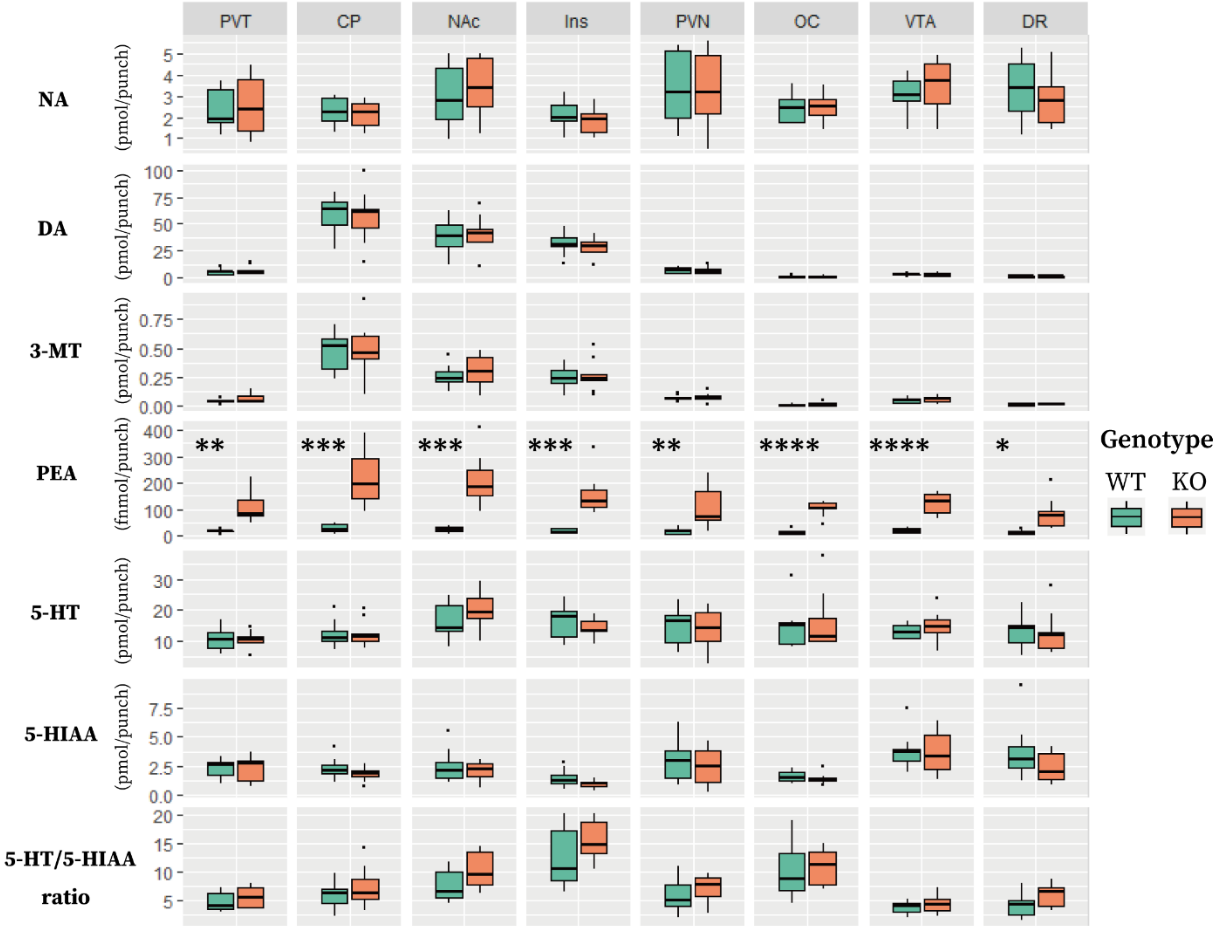

Supplementary Fig. S4: Brain MAs and metabolites in global *Maob* KO mice. The amount of substances in each brain region of wild-type (WT, *Maob*(+/Y)) and *Maob* KO (KO, *Maob*(−/Y)) mice (15–30-weeks old; n = 9 for each group). The amount of PEA is described as fmol/punch, and the others are described as pmol/punch. Boxplots show five statistics: median (horizontal bar), first and third quartiles (lower and upper hinges), and largest and smallest values no further than the 1.5-fold interquartile range (upper and lower whiskers). Data

beyond the the 1.5-fold interquartile range are plotted individually as outliers. Two-way ANOVA with the main effects of genotype and substance was applied in each brain region. The results for the PVT are also shown in Fig. 3b. All the other regions showed a significant difference depending on the genotype × substance interaction (CP: $df = 6$, $F = 28.16$, $P < 2\times10^{-16}$; NAc: $df = 6$, $F = 26.85$, $P < 2\times10^{-16}$; Ins: $df = 6$, $F = 28.54$, $P < 2\times10^{-16}$; PVN: $df = 6$, $F = 14.55$, $P = 3.21\times10^{-12}$; OC: $df = 6$, $F = 65.49$, $P < 2\times10^{-16}$; VTA: $df = 6$, $F = 53.25$, $P < 2\times10^{-16}$; and DR: $df = 6$, $F = 12.31$, $P = 1.33\times10^{-10}$). *$P < 0.05$, **$P < 0.01$, ***$P < 0.001$, ****$P < 0.0001$, Student's *t*-test with Bonferroni correction in each brain region. No significant difference in the NA, DA, 3-MT, 5-HT, 5-HIAA, or 5-HT/5-HIAA ratio between WT mice and *Maob* KO mice was found. DA, dopamine; DHBA, dihydroxybenzylamine; 5-HT, 5-hydroxytryptamine; 5-HIAA, 5-hydroxyindole acetic acid; NA, noradrenaline; 3-MT, 3-methoxytyramine; PEA, phenethylamine; CP, caudate-putamen; NAc, nucleus accumbens; Ins, insular cortex; PVN, paraventricular hypothalamic nucleus; OC, occipital cortex; VTA, ventral tegmental area; DR, dorsal raphe nucleus.

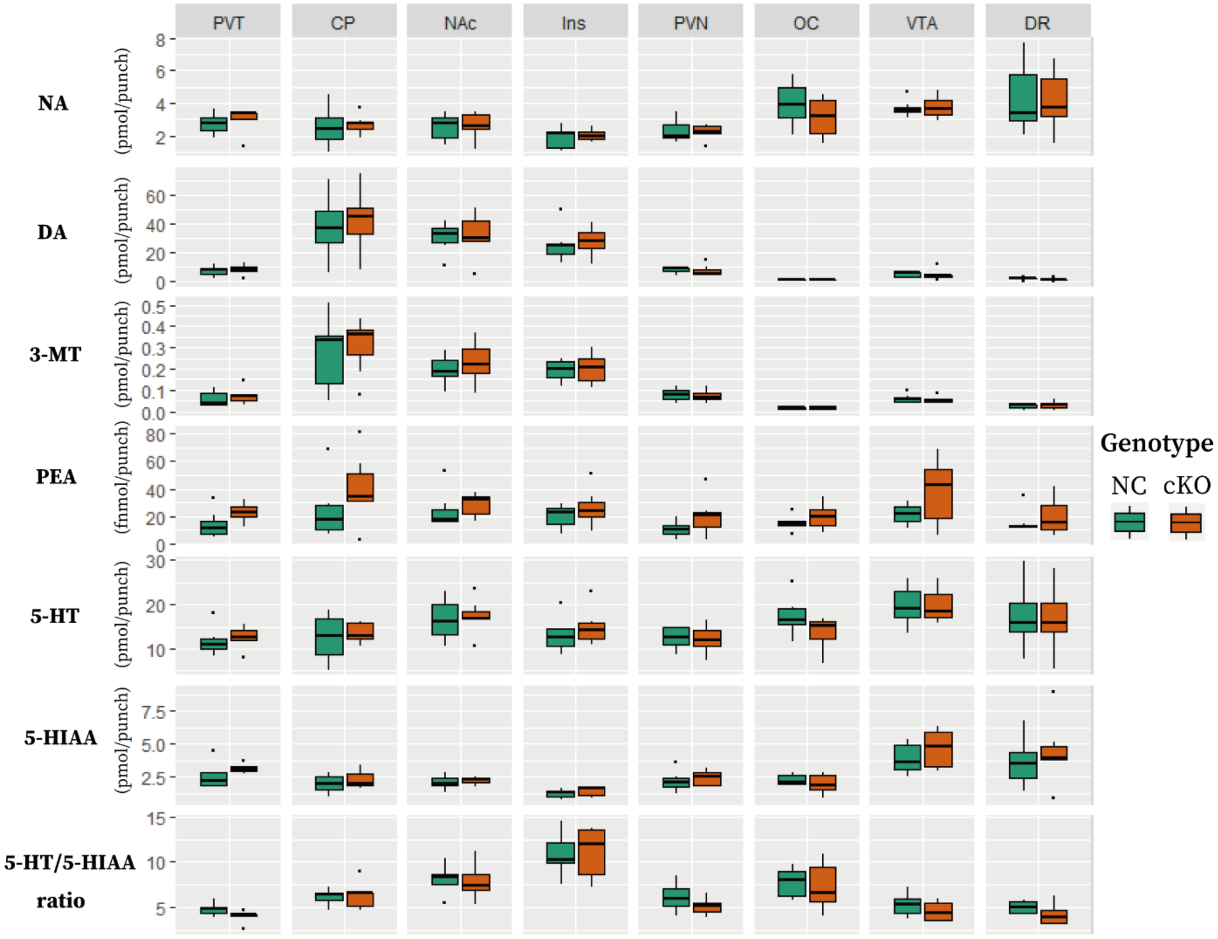

Supplementary Fig. S5: Brain MAs and metabolites in brain-specific *Maob* KO mice. The amount of substances in each brain region of control *Nestin*-Cre (NC, *Maob*(+/Y);*Nestin*(Tg/+)) and brain-specific *Maob* KO (cKO, *Maob*(flox/Y);*Nestin*(Tg/+)) mice (15–30-weeks old; n = 7 for each group). The amount of PEA is described as fmol/punch, and the others are described as pmol/punch. Boxplots show the same statistics as those shown in Supplementary Fig. S4. Two-way ANOVA with the main effects of genotype and substance was applied in each brain

region, revealing a significant difference depending on the genotype × substance interaction in the PVT (genotype: *df* =1, *F* = 4.290, *P* = 0.0414; substance: *df* = 6, *F* = 44.192, $P < 2\times10^{-16}$; and genotype × substance interaction: *df* = 6, *F* = 2.753, *P* = 0.0172). The genotype × substance interaction by two-way ANOVA in the other regions was as follows: CP: *df* = 6, *F* = 0.848, *P* = 0.537; NAc: *df* = 6, *F* = 0.167, *P* = 0.985; Ins: *df* = 6, *F* = 0.503, *P* = 0.804; PVN: *df* = 6, *F* = 2.449, *P* = 0.0313; OC: *df* = 6, *F* = 1.890, *P* = 0.0919; VTA: *df* = 6, *F* = 2.728, *P* = 0.018; and DR: *df* = 6, *F* = 0.396, *P* = 0.880. Student's *t*-test with Bonferroni correction in each brain region showed no significant increase in any substance in cKO mice. PEA, phenethylamine; CP, caudate-putamen; NAc, nucleus accumbens; Ins, insular cortex; PVN, paraventricular hypothalamic nucleus; OC, occipital cortex; VTA, ventral tegmental area; DR, dorsal raphe nucleus.

Supplementary Table S1: The filtering process applied to whole-exome sequencing data using the first-selection annotation data.

Selection of "novel" variants (not registered in dbSNP)
Exclusion of missense variants
Exclusion of variants detected by sequencing reads <10
Selection of variants not detected in the control wild-type C57BL/6J mouse
Exclusion of variants with allelic counts in the control wild-type C57BL/6J mouse >1
Selection of the variants with frequencies >0.25
Selection of variants detected in either the *Maob* KO or *Maob* flox mouse (not shared by the two lines)

Supplementary Table S2: PCR primers for reverse transcription-quantitative PCR.

Target gene	Forward	Reverse
Maoa	GCTACATGGAAGGTGCAGTTG	GGTGTGGGTAATTTCAAGAGC
Maob	GGCTGCTACACAACCTACTTCC	CAGTGTGAGGCTGTTTCAGTG
Ndp	CCGATCACAGCAGTATGTGC	CATTTGGAAGTAACAGGACCTC
Calb2	TCAGAAGAGTTCAATGCCATC	CTTCCTGTAGGTGGTGAGCTG
Actb	ATCACTATTGGCAACGAG	GGCATAGAGGTCTTTACGG
Gapdh	TGCGACTTCAACAGCAACTC	ATGTAGGCCATGAGGTCCAC

Supplementary Table S3: Multiple reaction monitoring transitions for quantification of target MAs and metabolites.

Substances	Ion mode	Precursor (m/z)	Product (m/z)	Retention time (min)
NA	Positive	152	135	0.99
DA	Positive	154	137	1.65
3-MT	Positive	168	151	3.07
PEA	Positive	122	105	3.36
5-HT	Positive	177	132<br>160	3.12
5-HIAA	Positive	192	91<br>146	3.83

Supplementary Table S4: Parameter settings used for LC-MS/MS analysisy of MAs and metabolites.

Detection algorithm	ICIS
Peak detection strategy (Analyte)	Nearest RT
Peak detection strategy (RT reference)	Nearest RT
Peak threshold type	Area
Threshold	1
Smoothing	1
Extracton window (min)	1.50
Area noise factor	1
Peak noise factor	3
Baseline window	40
Min peak height (S/N)	3.00
Noise method	Incos
Min peak width	3
Multiplet resolution	10
Area tail extension	5
Area scan window	4 or 6*

*When analyzing NA and DA in brain regions, the area scan window was set to six. All other analyses were performed with an area scan window of four.
